# Protocol for the systematic review of age and sex in preclinical models of age-correlated diseases

**DOI:** 10.12688/f1000research.153466.2

**Published:** 2024-11-25

**Authors:** Kai Diederich, Matthias Steinfath, Alexandra Bannach-Brown, Bettina Bert, Daniel Butzke, Paul Lucas Wildner, Maximilian Wurm, Ines Schadock, Céline Heinl

**Affiliations:** 1 German Centre for the Protection of Laboratory Animals (Bf3R), German Federal Institute for Risk Assessment (BfR), Berlin, Berlin, Germany; 2 QUEST Center, Berlin Institute of Health at Charité - Universitätsmedizin Berlin, Berlin, Berlin, Germany; 3 Charite University Hospital, Berlin, Germany

**Keywords:** systematic review, experimental animals, age, sex, translation

## Abstract

The translation of animal-based biomedical research into clinical research is often inadequate. Maximizing translation should be central to animal research on human diseases, guiding researchers in study design and animal model selection. However, practical considerations often drive the choice of animal model, which may not always reflect key patient characteristics, such as sex and age, impacting the disease's course. Despite diseases affecting both sexes, researchers frequently use male mice. To address this imbalance, journals and funding agencies have begun questioning the sex of animals used in studies and issued new guidelines. Conversely, the age of rodents is rarely discussed, even though many diseases primarily affect older patients. Young mice are commonly used, even in studies of diseases affecting older adults. Systematic comparisons between the age of rodents used and the age of patients in clinical trials are lacking. In this review, we systematically analyze the age and sex of mice used to model the five leading causes of global disability-adjusted life-years over the age of 75. We compare the results with the age and sex of patients in clinical trials focusing on Alzheimer's disease, stroke, type 2 diabetes mellitus, ischemic heart disease, and chronic obstructive pulmonary disease. We also analyze whether the age of the mice used has changed over the past decade. By systematically assessing the age and sex of the mice, we aim to initiate a discussion on the appropriate choice of animal model to improve the translatability of research results.

## Introduction

Animal research is primarily concerned with the study of human diseases and the search for potential treatments. ^1^ As the translation of basic research to humans is its main purpose, we would assume that maximizing the translational value of the results for patients should be central. Successful translation requires the validity of preclinical studies, both internal and external. Internal validity refers to experimental reproducibility, and external validity refers to the generalizability of results beyond a single research study. The external validity of preclinical studies is key for reflecting factors important for clinical translation, such as sex, age, and immune status. ^2^ Therefore, biological factors such as sex and age of the patients should be considered when choosing the experimental animal model, as they may influence the onset and course of a disease. However, sex is still not adequately represented in animal models of disease. ^3^ Until recently, it was assumed that the inclusion of female animals was not relevant in studies beyond the reproductive system and that the estrous cycle would add more unwanted variability. ^4^ This misconception has led to a preference for male mammals in many areas of research. ^5^ This concept has been refuted by evidence that sex affects most phenotypic traits in mice and that including both sexes lead to more robust and generalizable results. ^6^^–^^8^ Key players in the research system have since attempted to correct this imbalance by issuing new guidelines. ^9^^,^^10^ Funders such as the National Institutes of Health (NIH) now require researchers to justify using only one sex. In addition, several biomedical journals have adopted the Sex And Gender Equity in Research (SAGER) guidelines in their editorial policies, which require similar explanations. ^11^^,^^12^ An initial evaluation ten years after the introduction of these measures revealed a change toward a reduction in sex bias. ^3^ However, this improvement has not been uniform across all research fields. In neuroscience, for example, there was a large increase in studies involving both sexes, whereas in pharmacology, no change was observed. ^3^

An additional, but less discussed, biological factor is the representation of age in experimental animal models. Indeed, many diseases are age-related, and the age of patients plays an important role in the development and course of the disease. ^13^ However, the choice of rodent age may not always reflect the age of patients. In fact, a survey of nearly 300 researchers from different fields identified practical factors such as cost, animal supply or comparability with historical data as key factors. In this survey, the age of the rodents used, as reported by the respondents, clustered approximately 8-12 weeks for both mice and rats, regardless of the disease model being studied. ^14^ An age of 8-12 weeks in rodents corresponds to a young adult in humans. ^15^ Whether this age bias is observed in the broader scientific literature and whether it remains constant over time remain to be demonstrated.

In our planned systematic review, we will systematically assess the age and sex of mice in studies that focus on age-related diseases. We focus on mice because they are the most commonly used species in basic research on human diseases. We selected research areas according to the most recent assessment of the global burden of disease in 2019, published in 2020. ^16^ Thus, we will consider studies on the five leading causes of global disability-adjusted life years over the age of 75, namely, Alzheimer's disease, stroke, diabetes mellitus type 2, ischemic heart disease (IHD), and chronic obstructive pulmonary disease (COPD). As the last decade has been marked by extensive discussions on the reproducibility and translation of animal research, we will also assess whether the age of the mice used has changed in the last 10 years. Clinical trials are also characterized by an age bias toward younger patients compared to the general patient population. Therefore, we will also assess the age of patients enrolled in clinical trials for the five diseases studied for comparison with preclinical studies. ^17^ We do not plan a direct numerical comparison but rather determine which part of a life stage the mice are in compared to humans. In fact, some important biological processes can be identified in mice and humans that provide a framework that allows parallels to be drawn between the age of both species. ^18^ Thus, the mean duration of weaning period, puberty, adulthood, reproductive senescence and post senescence is known for mice and humans.

## Methods

We designed the study using the SYRCLE (Systematic Review Centre for Laboratory Animal Experimentation's) protocol template developed for systematic reviews of animal studies and the PRISMA-P checklist. ^19^^,^^20^ This protocol was registered at PROSPERO (CRD42023481414). To date, we have completed preliminary searches, conducted pilot studies to inform our statistical planning, tested our extraction process and started abstract screening.

### Research question

What is the age and sex of mice versus humans in published research of the age-correlated diseases: Alzheimer’s disease, stroke, diabetes mellitus type 2, IHD and chronic obstructive pulmonary disease? Our research question was based on the PICo scheme: **P**opulation (mice and humans), phenomenon of **I**nterest (age and sex) and **Co**ntext (age-correlated diseases). ^21^ We will further investigate whether the age and sex of the mice used in these mouse models have changed in the last ten years.

### Eligibility criteria

We defined different sets of inclusion and exclusion criteria for preclinical and clinical studies. For the preclinical literature, beyond the age and sex of the mice used, we will extract additional data, such as the animal model of the disease and the risk of bias. By sampling these data from the years 2023 and 2013, we want to evaluate the current state of the preclinical literature of the different fields and potentially detect a change in these factors within the last ten years. In contrast, for clinical studies, we only want to obtain an estimate of the age and sex of patients participating in clinical trials of specific diseases to make a narrative comparison to the age and sex of the animals. Based on these two main aims, we defined two different sets of eligibility criteria:

Preclinical research

We will include all studies designed to primarily investigate Alzheimer’s disease, stroke, diabetes mellitus type 2, IHD or COPD, independent of their study design. We will exclude studies that focus on other diseases.

We will only include studies using mice (genus Mus) *in vivo* or *ex vivo* to investigate one of the five considered diseases. Since it is not possible to translate and combine the ages of different animal species in a reasonable way, we decided to focus on mice, the most frequently used animal model. ^22^ Studies using other animals or cell lines will be excluded.

Studies will be included independently of the assessed outcome. However, studies that do not report the age of the mice will be excluded from the data analysis of age during full-text screening. In the second step, an excluded study due to missing age reporting will be replaced by another study to reach the predefined number of articles ( Figure 3).

We will only consider original articles and exclude abstract-only publications and reviews. To assess whether we see a change in the published literature within the last ten years, we will only use studies published either in 2013 or in 2023.

We will include studies in all languages with help from international collaborators and automated translation software.

Clinical research

We will use the Cochrane Library for the assessment of the age of patients included in the clinical trials. We selected Cochrane reviews because the authors extracted the age and sex of the included patients from the original trial reports that were retrieved upon systematic searches of different databases. We will only include Cochrane reviews that included studies with patients with one of the five investigated diseases. Only reviews including studies performed on healthy volunteers will be excluded.

Reviews will be included independently of the assessed outcome. However, if the entire Cochrane review does not report the age of the patients, this review will be excluded during full text screening and will be replaced by another review to reach the predefined number of articles. If only individual trials listed in a Cochrane review do not report age, the entire review will still be included.

We will only consider studies listed in Cochrane reviews. We will not exclude any publication year since the year of the clinical trials varies in any case within the Cochrane review, and we are not planning to analyze changes in the age or sex of patients over time. All the Cochrane reviews are all written in English; therefore, no language restrictions apply.

### Bibliographic search

Based on our different eligibility criteria for preclinical and clinical research, we will use different databases and bibliographic search strategies for preclinical and clinical studies.

Preclinical research

For the screening of the literature on research performed on mice, we will use Medline via PubMed® via and Embase via Ovid. Our search string will consist of medical subject headings (MeSH terms) for PubMed® and Emtree for Embase as well as keyword entries combined with Boolean operators. Our search string combines terms from four categories: animal, specific disease, year of publication and type of publication (see supporting information).

Clinical research

For the assessment of the age and sex of participants in clinical trials, we will use the Cochrane Library to search for eligible reviews. Since the database only includes reviews of human trials, we do not need to implement search strings for the species or the type of publication. We do not aim to analyze any change over time for the age of the patients and therefore do not restrain our search by publication date. Our search string therefore only includes synonyms for the specific diseases. We will use a combination of MeSH terms for the diseases as well as direct entries (see supporting information).

### Pilot study

Since the chosen diseases are research intensive, we are not aiming to provide a complete overview of the scientific literature of these fields. We rather want to get a valid estimate of the age and sex of the mice used and evaluate whether there has been a change in this regard in the last 10 years. Additionally, we are planning for a meaningful estimate of the age and sex of the patients included in clinical trials of the specified diseases to make a narrative comparison with the age of the rodents. To determine how many publications need to be screened for these two purposes, we performed two different pilot studies: one on the preclinical literature and one on the clinical literature.

Pilot study for preclinical literature screening

**Figure 1.**
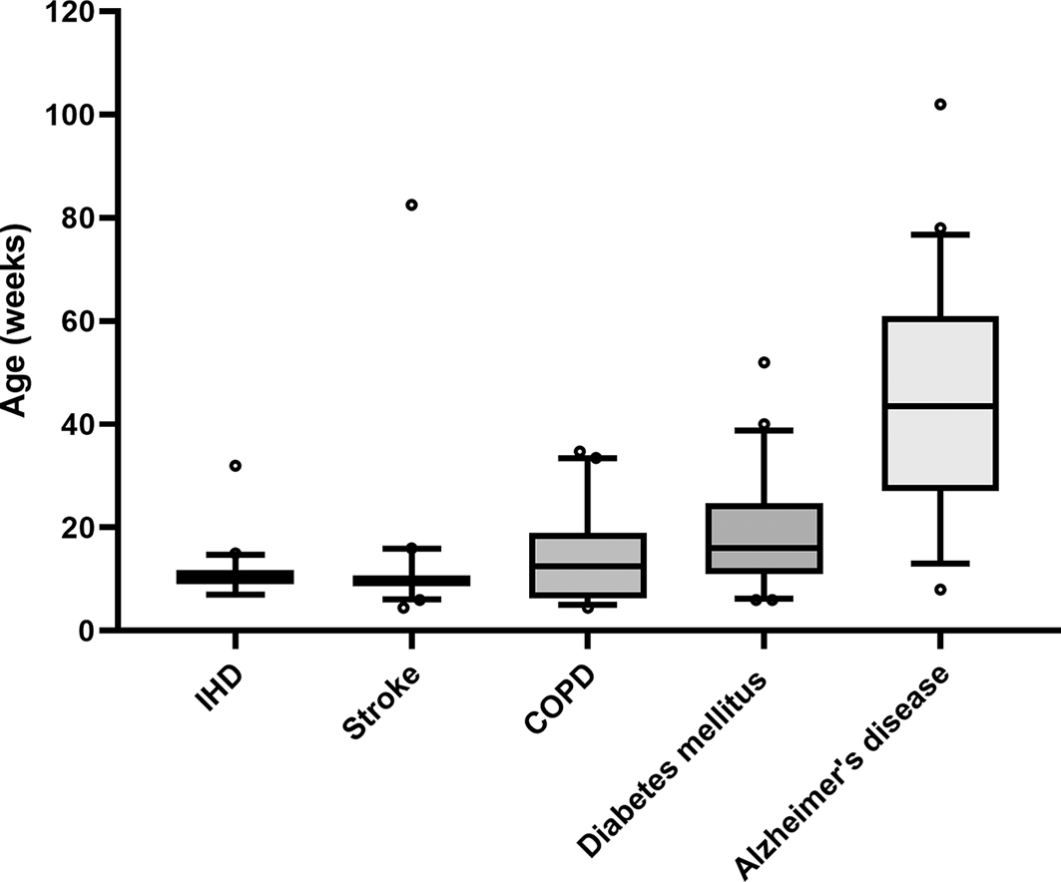
Age of mice extracted from 20 studies per disease that were randomly chosen. Whiskers denote the 10 ^th^ and 90 ^th^ percentiles, the central line represents the median, and o symbolizes outliers defined as more than 1.5 times the interquartile range.

The aim of the pilot study was to estimate the mean and variability of the age of the mice used in the different research fields. With these results, we have determined the number of articles that have to be screened to evaluate a change in the age of the mice within the last ten years. We therefore randomly selected 20 articles per disease of interest that were published within the last ten years. For this purpose, we performed a rough search in PubMed® with the names of the disease and the term “mouse”. We then created 20 random numbers within this range of results. We always started with the random number and then took the first article that met our eligibility criteria described above. From these articles, we extracted the ages of the mice used (see data extraction). Based on these results, we calculated the number of studies required to detect a biologically meaningful change in the age of the animals in the last ten years with a power of 80 % (Supporting information). Since the median and the variability of the ages of the mice differ greatly among the different diseases, we defined different age changes to be detected in the different diseases. First, a change of two weeks is biologically more meaningful in adolescent mice than in geriatric mice, and second, the number of studies that can realistically be screened is also restricted by the limited number of collaborators. Although the ages of the mice in the IHD and stroke groups strongly clusters around ten weeks without any notable variability, the mice in the COPD and diabetes mellitus type 2 research are older, and the variability is higher ( Figure 1). Research on Alzheimer’s disease seems to use the oldest mice and shows great variability. We therefore decided to aim for a detection of a difference of two weeks for IHD and stroke, three weeks for COPD and diabetes mellitus type 2 and eight weeks for Alzheimer’s disease ( Table 1). Since it could be hypothesized that some studies used very young mice and some old mice, the age distribution in the different studies might be multimodal. We therefore tested for this heterogeneity but could not detect any polymodality (Supporting information). This procedure will also be applied in the main study.

**Table 1.** Number of studies required to assess relevant changes in the age of mice between 2013 and 2023 based on a pilot study with 20 randomly selected studies per disease. Based on the median age, variability, and work force, we decided on an age difference that we would consider biologically relevant. We then calculated the number of studies required to detect this difference with a power of at least 80% (Supporting information).

	IHD	Stroke	COPD	Diabetes mellitus type 2	Alzheimer’s disease
** Median age in weeks**	10.50	10.00	12.50	16.00	43.50
**Age difference to be detected in weeks**	2	2	3	3	8
**Power**	0.80	0.81	0.80	0.80	0.81
**Number of studies required**	25	38	99	147	250

Pilot study for the clinical literature screen

**Figure 2.**
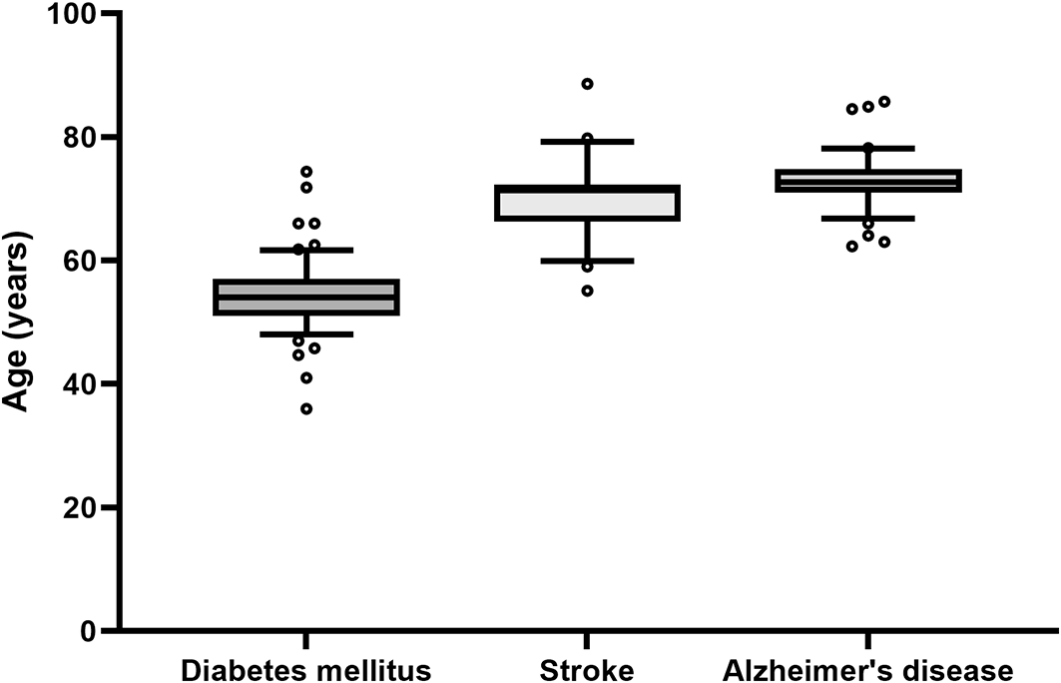
Boxplots of the age of patients included in the clinical trials listed in five Cochrane reviews of the respective disease. Although the median age varies between the diseases, the variability remains similar. Due to the variable number of trials per Cochrane review, each boxplot represents a different number of trials: Alzheimer’s disease: n = 46 (from 65 trials, 19 did not report the age of the patients); stroke: n = 27 (from 30 trials 3 did not report the age); diabetes mellitus type 2: n = 61 (from 123 trials 62 did not report the age). Whiskers denote the 10th and 90th percentiles, the central line represents the median, and o symbolizes outliers defined as more than 1.5 times the interquartile range.

To evaluate the age and sex of participants included in clinical trials, we will use a straightforward approach by using the extracted age and sex of patients from clinical trials that are listed in Cochrane reviews. In a pilot extraction, we assessed the median and the variability of the age of patients in clinical trials in a sample of five Cochrane reviews for three diseases each. We looked at Alzheimer’s disease and diabetes mellitus type 2, where we expected the strongest differences in age and variability and additionally included stroke as a third disease that we had anticipated to be between the other two. Indeed, whereas diabetes mellitus type 2 can occur at an earlier age, Alzheimer’s disease is mainly found in elderly patients. Accordingly, we found a median age of 54 years for patients with diabetes mellitus type 2, 72.65 years for patients with Alzheimer’s disease and 71.4 years for patients with stroke ( Figure 2). We did not observe meaningful differences in the variability between these three diseases and anticipate similar results for COPD and IHD. The included Cochrane reviews included different numbers of trials, resulting in different sample sizes for each disease. Thus, the five Cochrane reviews resulted in 61 trials for diabetes mellitus type 2, 27 trials for stroke and 46 trials for Alzheimer’s disease.

After assessing the variability in age within the trials included in one Cochrane review and between different reviews, we projected that analyzing ten Cochrane reviews for each considered disease would be precise enough to provide a valid estimate of the age of the patients included in clinical trials. In contrast to preclinical studies, we do not test for any change in time and therefore did not perform a power calculation. A detailed description of the method can be found in the supporting information.

### Random selection

**Figure 3.**
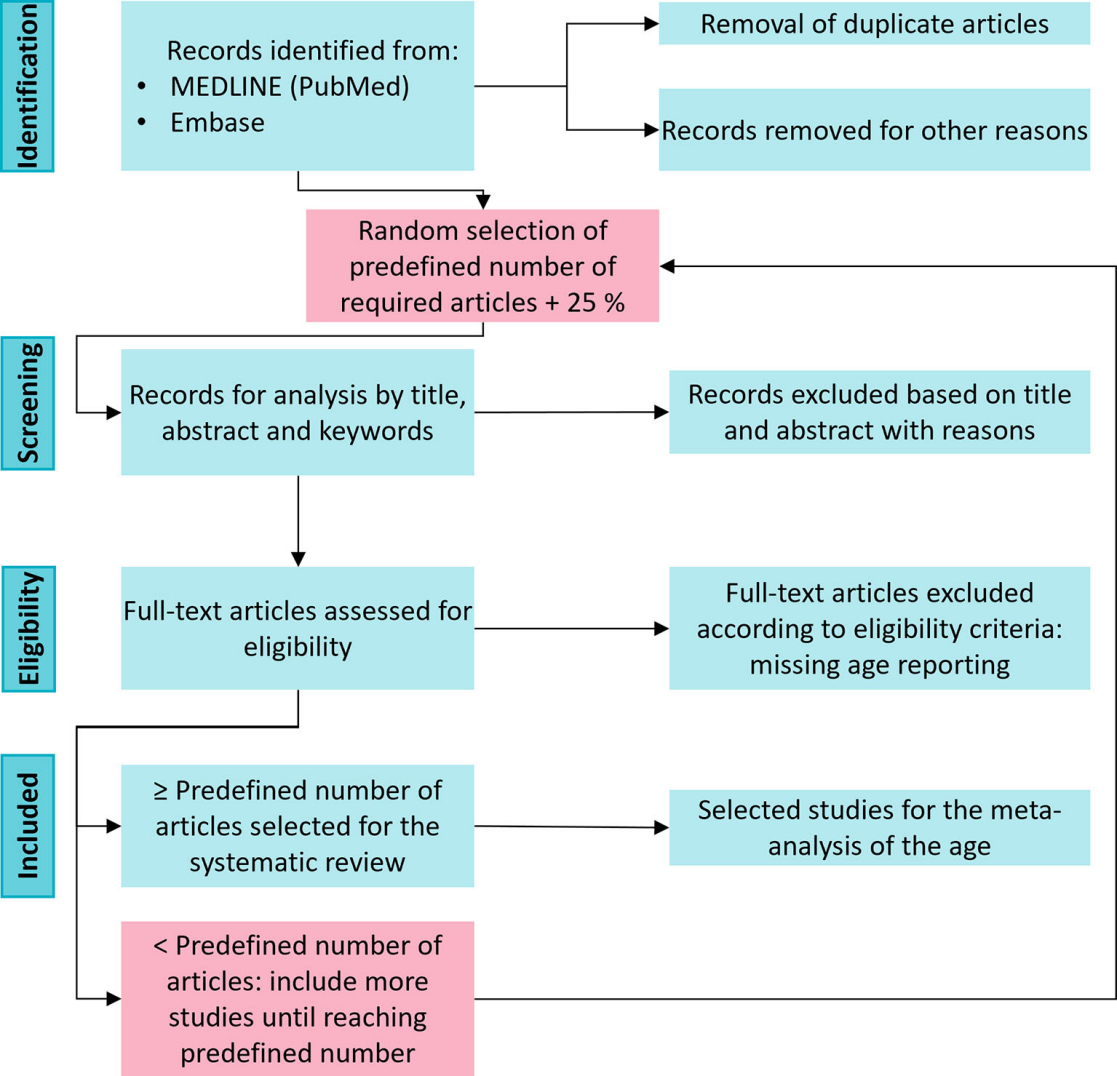
Flowchart of the eligibility of included preclinical studies. After retrieving all records and removing the duplicates, we will perform a random selection that will enter the screening phase. If, in the end, we do not reach the predefined number of required articles, we will fill this gap with a new random selection of studies.

Based on our pilot studies, we calculated the number of articles to be screened in advance. To select the required studies, we will import all the retrieved citations from our search from PubMed®/Medline and EMBASE for each disease individually into EndNote™. After removal of duplicates, we will generate a set of random citations with EndNote™. ^23^ We will use as many citations as predefined by our statistical planning plus an additional 25 % to account for dropouts for the full-text screening ( Figure 3). Our pilot screening revealed that approximately 25% of the studies did not report the age of the mice. The citations will be imported into SYRF (RRID:SCR_018907) for formal abstract screening. ^24^ If we do not reach the target number of articles after the full-text screening, we run the two screening phases again with an additional set of randomly selected citations.

Similarly, for the clinical studies, we will import all the search results from the Cochrane Library into EndNote™. After removal of the duplicates, we will perform a random pick and replace drop-outs until we reach the predefined number of Cochrane reviews in the same manner as described for the preclinical literature.

### Screening

Two independent reviewers, blinded to each other's scoring, will screen titles and abstracts against the inclusion and exclusion criteria. A third reviewer will resolve any discrepancies. Similarly, two independent reviewers, blinded to each other's assessments, will evaluate the full texts to determine final inclusion. If differences occur in the data extraction, a third reviewer will resolve divergences.

### Data extraction

We will retrieve the following information from the animal studies: bibliographic details, animal model characteristics, reporting quality and study quality. For clinical trials, we will only retrieve bibliographic details and patient characteristics. Since the studies included in a Cochrane review might already have been selected based on the study quality, the studies used do not reflect the general quality of the clinical literature for these research fields and will therefore not be extracted.

### Bibliographic details

Preclinical studies

We will extract the title, year of publication, first and last names of the first and last authors, and country of the institution of the first and last authors. Furthermore, for all studies included, we will define the probable gender of the first and last authors by combining the first name and the country of the institution. By assessing the probable gender of the first and last authors, we will analyze whether the gender of the researchers affects sex bias in preclinical and clinical studies. Indeed, a recently published study indicated that sex-related questions are more likely to be studied if the first or the last author of a publication is female. ^25^ However, whether this effect also affects the sex of the mice used in this research remains unknown. We will use the Gender API ( https://gender-api.com/de) for an automatic assessment of authors’ probable gender. The API provides an accuracy score for each first name in combination with the country of the institution where the study is performed.

Clinical studies

For the human studies, we will retrieve the title, the year of publication and the name of the authors and the country of the institution from the last author of the Cochrane reviews, as well as for the included original studies.

### Animal model characteristics and characteristics of the human patients

Preclinical studies

We will extract the age of the mice at the first outcome measurement after disease induction. If an age range is given, we will use the mean between the minimum and the maximum age. If only weight is given, we will convert it into age in weeks using information provided by animal suppliers. If the age or weight is not reported, we will note this and use it as an indicator of reporting quality and extract all other information from the article. We will, however, add another article to reach the predefined number of articles for the calculation of the mean age of the mice used. If different age groups of mice are used in a study, we will extract the age of the oldest group.

We will further assess the sex of the mice used, the strain, the supplier and whether the animals are genetically modified. We will also extract the model of induction for the specific diseases and determine the presence of comorbidities.

Clinical studies

For the clinical trials, we will extract the mean age of all patients of the trial and the sex of the patients from the Cochrane review. We will not record the ages of the participants in the healthy control groups. We will further record the presence of comorbidities.

### Reporting quality

Preclinical studies

As indicators of the reporting quality, we will evaluate whether all the animal model characteristics have been reported and how this reporting changed over time in the preclinical literature. We will differentiate whether age was given as weight, as a range with its extent or as exact age. We will note whether the sex was reported and if the exact number of animals for each sex is described. We will also assess the level of accessibility of the study. We will note whether the article was published open access and whether the data were shared and categorize this accessibility. We define open access as green, gray or gold open access. For the data sharing, we will differentiate between those available for download from the journals’ website or a repository, those available upon request, those for which data sharing is not applicable (e.g., “all data appear in the article” or “no datasets were generated or analyzed”) and those for which no data availability statement is available.

Clinical studies

The studies included in the Cochrane reviews do not reflect the general clinical literature, as they might be selected based on quality criteria. Hence, we refrain from extracting these data.

### Study quality

Preclinical studies

To evaluate the study quality, we will use a modified version of the CAMARADES checklist for study quality. ^26^ We will thus extract the reporting of random allocation, allocation concealment, blinded outcome assessment, sample size calculation, animal exclusions (including reasons and number of excluded animals), potential conflicts of interest, and compliance with animal welfare regulations. However, quality will not be used as an eligibility criterion.

Clinical studies

Since the focus of our study is preclinical research, the quality of the clinical trials is out of scope and will not be further evaluated.

### Data synthesis

Preclinical studies

We will synthesize the age in weeks for each research field and for each year. We will compare whether we can detect a change in age between the ages of the mice used in publications in 2013 and those used in 2023.

Clinical studies

We will synthesize the age of the patients enrolled in the clinical trials of the specific diseases. We are not planning to statistically compare the age of the mice versus the age of the patients; however, after calculating the mean ages, we will make a qualitative comparison.

## Discussion

The translation of promising results from animal experiments in human patients is limited. There are multiple reasons for this unsatisfactory success rate. ^27^ Not only biomedical research but also other disciplines are facing a major reproducibility crisis. ^28^ The lack of reproducibility may be caused by factors unrelated to the biology of the model organisms, such as selective reporting of outcomes, poor statistical planning, and insufficient reporting. ^28^^,^^29^ However, in addition to these general problems that may occur across research fields, the poor reflection of patient characteristics in animal models and other threats to external validity might play a critical role in translational failure. ^30^ Our study cannot show any causal relationship between insufficient translation and the choice of animal model. However, we can identify and discuss discrepancies in biological characteristics between preclinical and clinical research.

Our statistical planning, including the sample size calculation for preclinical studies, is entirely based on comparisons of the ages of mice between different years. This means that other analyses, e.g., the reporting quality or the sex of the animals, will be exploratory. Our approach to using Cochrane reviews has some limitations, as Cochrane reviews already have some eligibility criteria for the included trials that might affect the age and sex of the patients. However, these methods are straightforward for obtaining an estimate of the age of the patients included in clinical trials and might also be affected by age bias. ^31^

We expect that with our systematic review, we can transparently show the extent of the age gap in preclinical biomedical research and initiate a broader discussion on the topic.

## Ethics approval and consent to participate

Not applicable

## Consent for publication

Not applicable

## Authors' contributions

KD, AB-B, BB, and CH: developed the study concept and design. KD, MS, DB, PLW, MW, IS, and CH were involved in data acquisition and analysis. KD and CH: drafted the manuscript and figures. All authors contributed to the article and approved the submitted version.

## Data Availability

All data associated with this article are available on the Open Science Framework (OSF). OSF: Systematic Review of Age and Sex in Preclinical Models of Age-Correlated Diseases. https://osf.io/zma35/ ^32^
• Prisma-P flowchart of the eligibility of included preclinical studies • Prisma-P Checklist • Data sheet: clinical pilot data • Data sheet: preclinical pilot data Data are available under the terms of the Creative Commons Attribution 4.0 International license (CC-BY 4.0). OSF: Systematic Review of Age and Sex in Preclinical Models of Age-Correlated Diseases. https://osf.io/zma35/ ^32^
• Supporting Information: search strings and evaluation of the pilot study for preclinical studies and sample size determination Github: Systematic review of age and sex in preclinical models of age correlated-diseases. https://github.com/celinebf3r/Systematic-review-of-age-and-sex-in-preclinical-models-of-age-correlated-diseases ^33^
• R-Code and data used and collected in the pilot study that served as the basis for the development of the study protocol • Data and code are available under the terms of the Creative Commons Attribution 4.0 International license (CC-BY 4.0).
