# Supplementary material for: Protocol for the systematic review of age and sex in preclinical models of age-correlated diseases
Source: F1000Res. 2024 Nov 25;13:858. Originally published 2024 Aug 1. [Version 2] doi: 10.12688/f1000research.153466.2 (PMC11747299; doi:10.12688/f1000research.153466.2)
Supplement: Peer review history

# Reviewer report: Reviewer response for version 2

**DOI:** 10.5256/f1000research.174899.r359399

**Published:** 2025-01-20

**Authors:** Alberto Granzotto

**Affiliations:** 1 University G. d’Annunzio of Chieti-Pescara, Chieti, Italy

In their work, Diederich et al. propose a detailed protocol for the systematic comparison of age and sex of mice used to model relevant human diseases, like Alzheimer's disease (AD), stroke, type 2 diabetes, ischemic heart disease, and chronic obstructive pulmonary disease.

 The study is timely as addresses an important topic at the intersection between basic and clinical research, with implications for translability, study design, and data interpretation. The rationale is clear and the proposed approach is well structured.

 I have only two minor technical points. One concerns the weight-to-age conversion using animal supplier provided information. This could be suboptimal considering potential differences in the chow diet used by different labs or the approaches employed for generating a disease phenotype (i.e.: T2DM). This point should be clarified.

 As a researcher actively involved in the AD field, the second point regards the model(s) included in the analysis since some of them recapitulate only the amyloid pathology, other only the tau pathology, very few both, although all are considered AD models. This point should be briefly elaborated.

 In addition, there is debate on the validity of mouse models in primarily human diseases like AD [1,2,3], including age- and sex-related issues. Potential specific caveats for the proposed study conditions should be (very briefly) discussed to provide more context.

Is the study design appropriate for the research question?

Yes

Is the rationale for, and objectives of, the study clearly described?

Yes

Are sufficient details of the methods provided to allow replication by others?

Yes

Are the datasets clearly presented in a useable and accessible format?

Yes

Reviewer Expertise:

Alzheimer's disease, neurodegeneration

I confirm that I have read this submission and believe that I have an appropriate level of expertise to confirm that it is of an acceptable scientific standard.

## References

[1] : Clinical relevance of animal models in aging-related dementia research. *Nat Aging* .2023;3(5) : 10.1038/s43587-023-00402-4 481-493 10.1038/s43587-023-00402-4 37202516

[2] : Lost in translation: Inconvenient truths on the utility of mouse models in Alzheimer's disease research. *Elife* .2024;13: 10.7554/eLife.90633 10.7554/eLife.90633 39329365 PMC11434637

[3] : Going beyond established model systems of Alzheimer's disease: companion animals provide novel insights into the neurobiology of aging. *Commun Biol* .2023;6(1) : 10.1038/s42003-023-05034-3 655 10.1038/s42003-023-05034-3 37344566 PMC10284893

# Reviewer report: Reviewer response for version 2

**DOI:** 10.5256/f1000research.174899.r343344

**Published:** 2024-12-04

**Authors:** Devin Wahl

**Affiliations:** 1 Colorado State University, Fort Collins, Colorado, USA

I have gone through the author’s comments, responses, and main text. I now believe that this article is suitable for indexing

Is the study design appropriate for the research question?

Yes

Is the rationale for, and objectives of, the study clearly described?

Yes

Are sufficient details of the methods provided to allow replication by others?

Partly

Are the datasets clearly presented in a useable and accessible format?

Yes

Reviewer Expertise:

NA

I confirm that I have read this submission and believe that I have an appropriate level of expertise to confirm that it is of an acceptable scientific standard.

# Reviewer report: Reviewer response for version 1

**DOI:** 10.5256/f1000research.168374.r327825

**Published:** 2024-10-16

**Authors:** Devin Wahl

**Affiliations:** 1 Colorado State University, Fort Collins, Colorado, USA

In the current analysis, Diederich et al. suggest to perform a systematic review of animal studies to assess the age and sex of mice used in basic research on age-related human diseases. The authors choose to focus on five major diseases (the leading causes of death worldwide). The authors will use The Systematic Review Centre for Laboratory Animal Experimentation’s protocol template and the PRIMSA-P checklist. Importantly, the authors suggest to perform these analyses at several timepoints (2013 and 2023), thus providing insight into how these factors have changed within the last 10 years. The authors also will examine clinical trials via the assessment of Cochrane reviews (also examining the five diseases as described above).

 Overall, the manuscript is well written and easy to follow. In my mind I feel as if this these analyses will make a good contribution to the literature (and they are important). I have a couple of suggestions for the authors that might improve their analyses and contributions to this field.

1. The authors might provide more of a discussion on why they chose the five diseases. They provide a general overview; However, some readers might also appreciate, for example, a discussion of cancer because aging is also a leading cause of many types of cancers. This is just an example. 
2. The analyses would benefit (or at least a discussion would be appropriate) by also considering different mouse models. For example, one would assume that most of these mice are C57/B6. Would the authors suggest different genetic models or genetically diverse mice, and to what extent should those be included in the analysis? More of a discussion is necessary as that would strengthen the manuscript.
3. How do the authors ‘compare’ the ages of mice and humans? In other words, how do the authors know that a six-months old mouse is comparable to a middle-aged human? It will be important o examine the literature on this.

Is the study design appropriate for the research question?

Yes

Is the rationale for, and objectives of, the study clearly described?

Yes

Are sufficient details of the methods provided to allow replication by others?

Partly

Are the datasets clearly presented in a useable and accessible format?

Yes

Reviewer Expertise:

NA

I confirm that I have read this submission and believe that I have an appropriate level of expertise to confirm that it is of an acceptable scientific standard, however I have significant reservations, as outlined above.

# Response

**Published:** 2024-11-15

**Authors:** Kai Diederich

**Affiliations:** German Federal Institute for Risk Assessment, Berlin, Germany

Dear Dr. Devin Wahl,

 thank you for your positive review and critical analysis of our manuscript. We would like to clarify the specific issues raised in the following.

*Comment 1: The authors might provide more of a discussion on why they chose the five diseases. They provide a general overview; However, some readers might also appreciate, for example, a discussion of cancer because aging is also a leading cause of many types of cancers. This is just an example.*

 Response: We thank the reviewer for this comment. We agree, that cancer is one of the major age-related diseases. Therefore, the question of selecting the most appropriate animal model for preclinical research also arises in this field. This will be discussed in detail in the manuscript of our meta-analysis. In order to include enough studies for each considered disease to ensure valid outcomes, we had to limit our review to a subset of diseases. These were selected according to the most recent assessment of the global burden of disease in 2019, published in 2020 (Global burden of 369 diseases and injuries in 204 countries and territories, 1990–2019: a systematic analysis for the Global Burden of Disease Study 2019, The Lancet, Volume 396, Issue 10258, 1204 – 1222). We chose to focus on the five leading causes of global disability-adjusted life years over the age of 75 in our systematic review, namely Alzheimer's disease, stroke, diabetes mellitus type 2, ischaemic heart disease (IHD), and chronic obstructive pulmonary disease (COPD).

*Comment 2: The analyses would benefit (or at least a discussion would be appropriate) by also considering different mouse models. For example, one would assume that most of these mice are C57/B6. Would the authors suggest different genetic models or genetically diverse mice, and to what extent should those be included in the analysis? More of a discussion is necessary as that would strengthen the manuscript.*

 Response: We thank the reviewer for this comment. We will include studies using mice (genus Mus) in vivo or ex vivo to investigate one of the five diseases under consideration. This encompasses both wild-type mice and genetically modified animals. This is a crucial consideration, for instance, when examining Alzheimer's disease models, as these predominantly comprise genetically modified animals. Additionally, the genetic model may influence the age of the animal. For instance, there are distinct transgenic animals for early-onset and late-onset Alzheimer's disease. Early-onset Alzheimer's is often associated with familial mutations in genes like *APP* (amyloid precursor protein), *PSEN1*(presenilin-1), and *PSEN2* (presenilin-2), while late-onset Alzheimer's is linked more to complex genetic factors, such as the presence of the *APOE* ε4 allele and mutations in genes like *TREM2*, which affect microglial response. Therefore, in addition to sex and age, we investigate genetic modifications, as well as race, the supplier, the induction model for the specific disease and the presence of comorbidities. This is described in the section ‘Characteristics of the animal model and characteristics of the human patients’. These parameters will be discussed in detail in the manuscript of our meta-analysis.

*Comment 3: How do the authors ‘compare’ the ages of mice and humans? In other words, how do the authors know that a six-months old mouse is comparable to a middle-aged human? It will be important to examine the literature on this*

 Response: Thank you for this very important comment. We have refrained from doing a direct numerical comparison. Since the duration of developmental phases in mice compared to humans are not uniformly correlated. It rather seems that especially the early life in mice is accelerated compared to humans (1). It is therefore not possible to simply take the absolute life span of humans and mice and to calculate a factor translating the age.

 However, some important biological processes can be defined in mice and humans and can provide a framework enabling drawing parallels in the age of both species. Thus, the mean duration of weaning period, puberty, adulthood, reproductive senescence and post senescence is known for mice and humans (1).  

 Since we expect a strong discrepancy between the age of mice included in research studies and the age of human patients in clinical studies of age-correlated disease, we probably will not need an exact translation of the age but rather determine which part of a life stage the mice are in compared to humans.

 We have now included this discussion in the introduction of the manuscript and also added a new reference.

 1.           Dutta S, Sengupta P. Men and mice: Relating their ages. Life Sciences. 2016; https://doi.org/10.1016/j.lfs.2015.10.025
